# Calcium phosphate mineralization in bone tissues directly observed in aqueous liquid by atmospheric SEM (ASEM) without staining: microfluidics crystallization chamber and immuno-EM

**DOI:** 10.1038/s41598-019-43608-6

**Published:** 2019-05-14

**Authors:** Chikara Sato, Daiju Yamazaki, Mari Sato, Hiroshi Takeshima, Nassirhadjy Memtily, Yuri Hatano, Takayuki Tsukuba, Eiko Sakai

**Affiliations:** 1 Biomedical Research Institute, National Institute of Advanced Industrial Science and Technology (AIST), Central 6, Higashi 1-1-1, Tsukuba, Ibaraki, 305-8568 Japan; 2 Graduate School of Pharmaceutical Sciences, and Graduate School of Medicine, Kyoto University, Yoshida Shimo Adachi, 46-29 Sakyo, Kyoto, 606-8501 Japan; 3 Traditional Uyghur Medicine Institute of Xinjiang Medical University, 393 Xinyi Rd, Xinjiang Uyghur Autonomous Region, Urumqi, 830011 China; 4 Division of Dental Pharmacology, Department of Developmental and Reconstructive Medicine, Nagasaki University Graduate School of Biomedical Sciences, 1-7-1 Sakamoto, Nagasaki, 852-8588 Japan

**Keywords:** Bone development, Targeted bone remodelling

## Abstract

The malformation and disordered remodeling of bones induce various diseases, including osteoporosis. We have developed atmospheric SEM (ASEM) to directly observe aldehyde-fixed bone tissue immersed in radical scavenger buffer without thin sectioning. The short procedure realized the observation of bone mineralization surrounded by many cells and matrices in natural aqueous buffer, decreasing the risk of changes. In osteoblast primary cultures, mineralization was visible without staining. Correlative energy dispersive X-ray spectrometry indicated the formation of calcium phosphate mineral. Fixed bone was sectioned, and the section surface was inspected by ASEM. Mineralized trabeculae of talus spongy bone were directly visible. Associated large and small cells were revealed by phosphotungstic acid staining, suggesting remodeling by bone-absorbing osteoclasts and bone-rebuilding osteoblasts. In tibia, cortical bone layer including dense grains, was bordered by many cells with protrusions. Tissue immuno-EM performed in solution for the first time and anti-cathepsin-K antibody, successfully identified osteoclasts in femur spongy bone. A microfluidics chamber fabricated on the silicon nitride film window of an ASEM dish allowed mineralization to be monitored *in vitro*; calcium phosphate crystals as small as 50 nm were imaged. ASEM is expected to be widely applied to study bio-mineralization and bone-remodeling, and to help diagnose bone-related diseases.

## Introduction

Mineralization plays a crucial role in life. As animals evolved, the formation of extracellular collagen conferred mechanical strength to multicellular organisms, allowing the size of their bodies to increase. Later, calcium phosphate (CaP) precipitation followed by oxidization formed hydroxylapatite (HA) on crosslinked collagen fibers, leading to bone formation. This resulted in further body enlargement, especially during the evolution of air-breathing vertebrates. Mineralization also serves as a calcium stock and plays significant roles in calcium metabolism. In human bodies, bones are constantly remodeled to maintain their structural matrix and strength; bone is partially absorbed by osteoclasts and rebuilt by osteoblasts in micro-remodeling pits^1^. The malformation and disordered remodeling of bones caused, in part, by the unbalanced activity of these cells, are related to diseases such as osteoporosis, osteopetrosis and osteoarthritis^2^.

The mineralization that takes place during bone development has been intensively studied by optical microscopy (OM) and electron microscopy (EM)^3,4^, but the methodological limitations of high-resolution microscopy for bone tissues make it difficult to study bone remodeling and mineralization. Transmission EM (TEM) of Epon-embedded specimens is widely employed^5^. While lightly mineralized tissues can be embedded and thin-sectioned without decalcification, densely mineralized tissues generally need to be demineralized (decalcified) beforehand^6^. This involves the use of EDTA for a relatively long period of time (typically 3 weeks). Further, Epon-embedding and thin-sectioning include hydrophobic treatments and take additional days, which together might affect mineralization and attached hydrophilic tissues. A new fixation method to preserve tiny precipitates under anhydrous conditions developed by Boonrengsiman *et al*., makes it possible to image the mineral granules less than 50 nm in mitochondria in osteoblasts by TEM^7^.

Quantitative backscattered electron imaging (qBSE/qBEI) scanning EM (SEM) techniques are generally used to examine non-decalcified, dehydrated, resin-embedded and polished thick sections of bone^8–10^. The measurement can differentiate between changes in bone volume or in the degree of mineralization of the bone matrix. Methods that allow bone to be observed without prior hydrophobic treatment are lacking.

Environmental capsules were developed to image wet samples for TEM^11–13^ and SEM^14^. Such systems have been successfully employed to image hydrophilic molecular complexes^15^, cells^16^ and tissues^17,18^. This development led to the first visualization of biological mineralization in wet conditions. Vidavsky *et al*. observed calcium carbonate mineralization in sea urchin embryos in wet conditions^19^ using a B-nano microscope (a field emission SEM (FE-SEM) column sealed by a SiN film at the bottom end)^20^. Until now, CaP mineralization has not been observed in liquid by EM.

**Figure 1:**
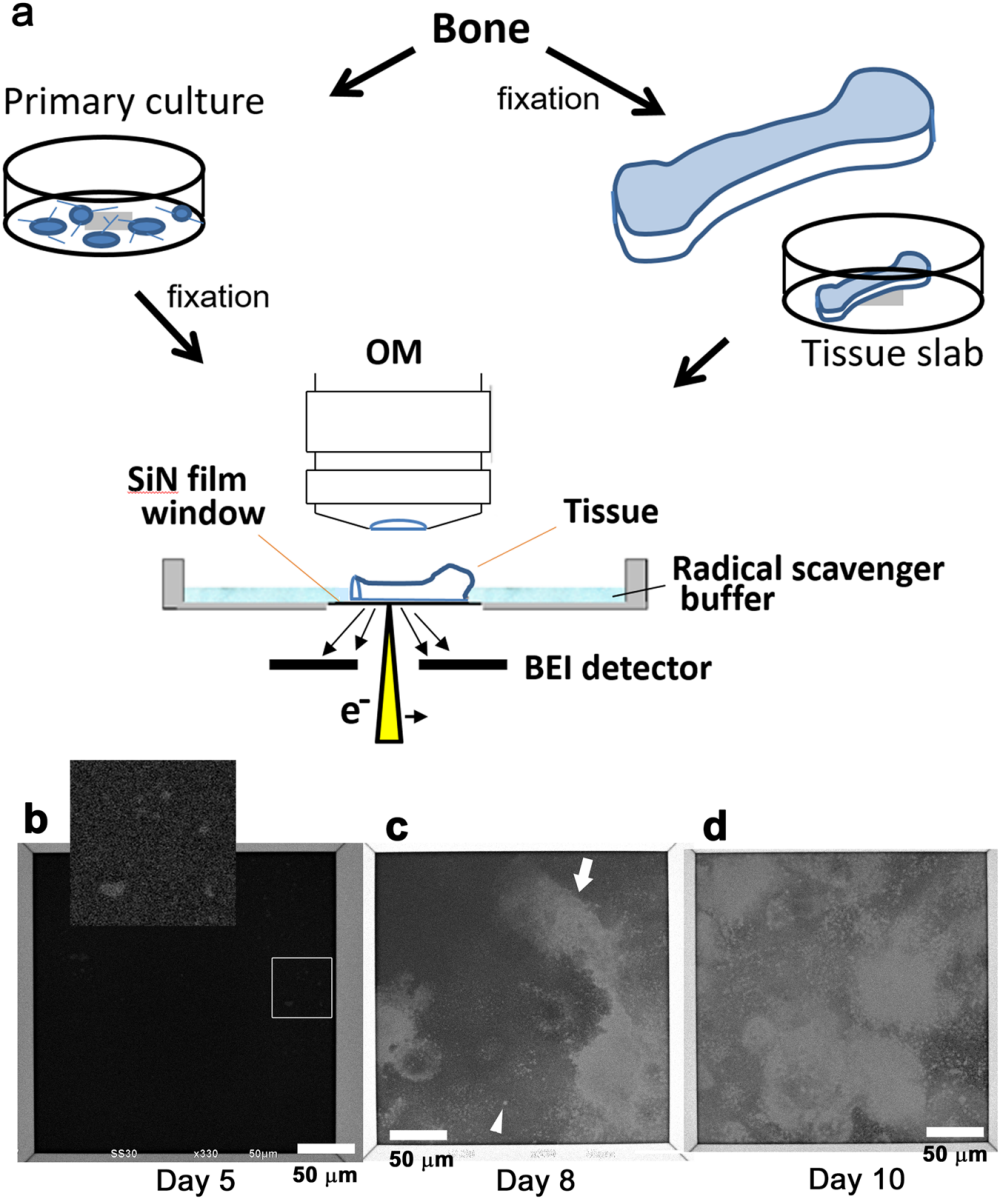
ASEM of unstained osteoblast primary culture or bone tissue immersed in aqueous liquid. (**a**) ASEM system used to image unstained osteoblasts or bone tissues. Tissue or cultured cells were aldehyde fixed and observed in radical scavenger glucose solution. In the ASEM system, an OM is positioned above an inverted SEM, with the specimen dish in between. The removable, 35-mm ASEM specimen dish is open to the atmosphere and has eight SiN windows in its bottom plate. The base of the ASEM dish seals the microscope chamber and separates the sample immersed in aqueous solution from the microscope vacuum. Each window is a 100 nm-thick SiN film (0.25 × 0.25 mm). Osteoblasts were cultured on the dish, fixed with GA and directly observed in solution. Fixed tissues were excised from the organ and placed on the dish with the excised face down, i.e., in contact with the SiN membrane. The electron beam projects from underneath onto the fixed cells or tissues through the SiN film, and backscattered electrons are detected by the BEI detector of the ASEM, allowing high resolution imaging. (**b**–**d**) Osteoblast culture observed over time by ASEM. (**b**) Day (DID) 5 after induction of differentiation, very small bright signals start to appear, indicating that electron-dense sedimentations begin to emerge. The contrast and brightness are adjusted in the enlarged subpanel in the top. (**c**) DID8; bright spots appear, forming cell shapes (arrow) surrounded by fine dots (arrowhead). (**d**) DID10; the number of bright spots increases as the culture proceeds. Scale bars: 50 μm.

In this paper, we report the use of our developed atmospheric SEM (ASEM)^21,22^ to investigate CaP mineralization in bone and *in vitro*. Based on protocols developed for other samples including neurons^23–25^, bacteria biofilm^26^, Langerhans islet in pancreas^27^ and cardiac- and skeletal- muscles^28^ bearing end plates^29^, the cells and tissues were aldehyde fixed, immersed in aqueous solution containing a radical scavenger (glucose) - to minimize the effect of radicals formed by electron radiation at low-accelerating voltage (30 kV) suppressing knock-on damage - and imaged by ASEM (Fig. 1). We successfully observed mineralization using osteoblast culture. We also observed fine and large mineralization in cortical and spongy bone tissues and the attached non mineralized cells and structures using staining with metal solution. Further, to identify osteoclasts, immuno-EM of tissue in solution was realized for the first time using anti-cathepsin K protease antibody.

## Results

### Mineralization process in primary cultured osteoblasts

To follow mineralization, mouse osteoblast primary cultures were imaged over time without staining using ASEM. Since CaP precipitates and the HA subsequently formed both have a high electron density (atomic numbers: Ca = 20, P = 15), we expected the process to be visible without staining, even in aqueous solution (atomic numbers: H = 1, O = 8). Osteoblasts from neonatal mouse calvaria were cultured on 8-window ASEM dishes^28^ in a CO_2_ incubator^30^, and at a given time point the cells in some dishes were fixed with glutaraldehyde (GA) and observed in radical scavenger glucose solution (Fig. 1a, left path). On culture day 5 (DID5) from induction of differentiation, slight signals started to emerge in some of the dishes (Fig. 1b, inlet). Mineralization onset varied from area to area on the same dish and from dish to dish: almost half of the ASEM dishes were not mineralized at this time-point. On DID8, bright patches indicating high electron density appeared, sometimes forming cell-like shapes (Fig. 1c and Supplementary Fig. 1a,b, arrows). The patches were surrounded by small bright spots (arrowheads). The number and intensity of the signals, which we assign to mineralization, generally increased as culture proceeded. The degree of mineralization varied from location to location (Fig. 1d and Supplementary Fig. 1c,d).

### Elemental composition analysis confirmed CaP mineralization

**Figure 2:**
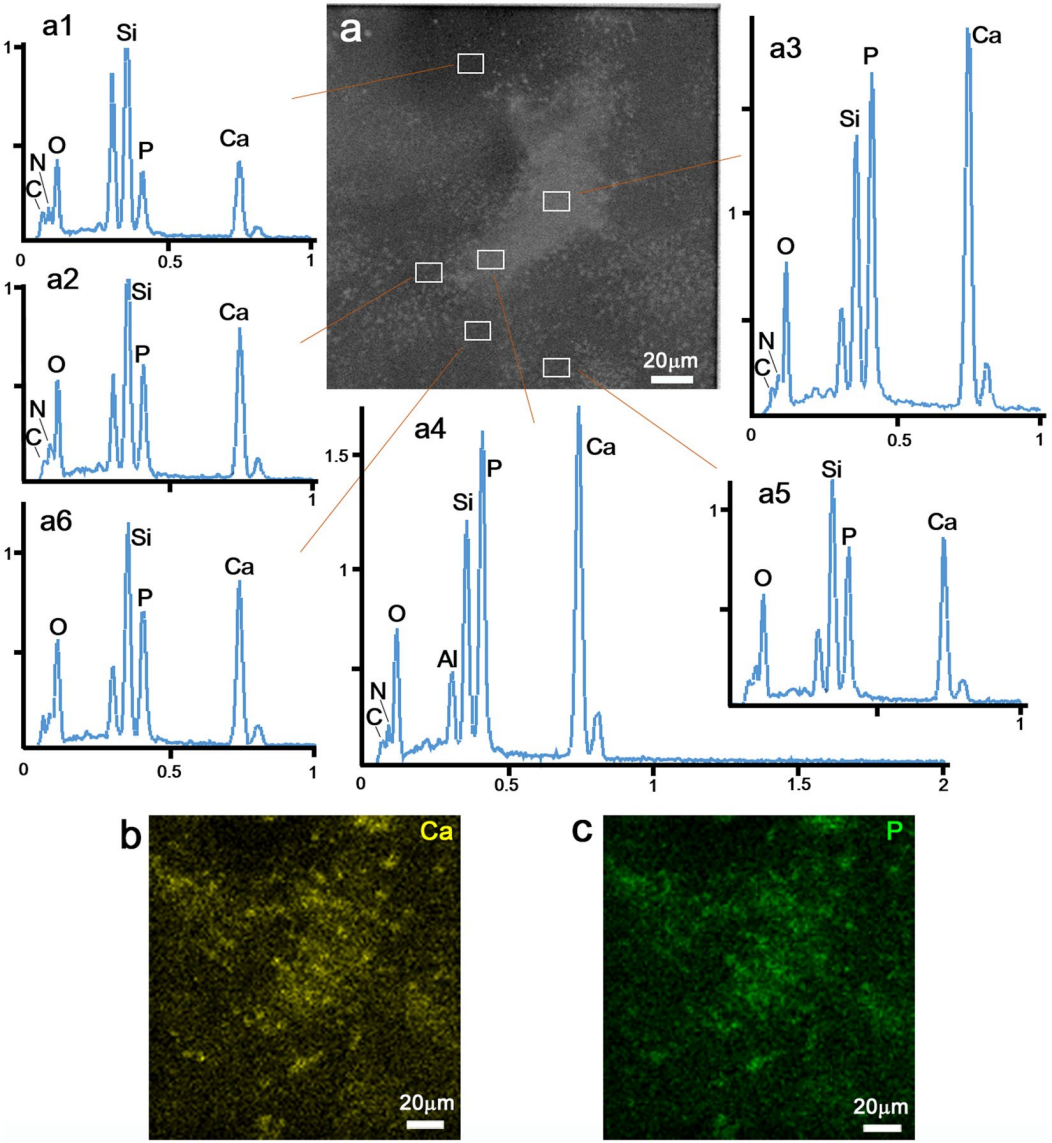
Element analysis of the electron dense signals. After ASEM imaging of GA-fixed osteoblast culture (DID10) in solution, the culture on the ASEM dish was progressively dehydrated using ethanol, dried, and placed in the vacuum chamber of an EDS FE-SEM. (**a**) ASEM image of GA-fixed ostoblast cells in solution. The indicated areas were examined from the top of the dish by EDS. (**a1**–**a6**) Typical EDS spectra recorded from the white square areas indicated in a. The major mineral components of bone, Ca, P and O, are more abundant in brighter areas of the ASEM image, suggesting that the BEI signals represent CaP mineral formation. A large amount of Si was always detected, reflecting the SiN film window underneath the cells. (**b**,**c**) Ca and P element distributions mapped using EDS. Both maps resemble the distribution of the electron-dense signals registered by ASEM (panel a).

To confirm that the electron-dense signals were CaP mineralization, cells cultured on the ASEM dish were GA-fixed, observed, then dehydrated using an alcohol series, dried and placed in the vacuum chamber of a standard FE-SEM equipped with an energy dispersive X-ray spectrometer (EDS). EDS spectra were recorded from various areas of the sample. Typical results are shown in Fig. 2. The two-dimensional localization of calcium and phosphate determined (Fig. 2b,c) correlates well with the bright signals observed by ASEM (Fig. 2a); bright areas in the ASEM image usually gave EDS spectra with large Ca and P peaks and darker areas EDS spectra with smaller Ca and P peaks, i.e., the Ca and P content increased as ASEM image brightness increased (Fig. 2a1–a6). The slight difference between the Ca and P localization maps (Fig. 2b,c) and the ASEM image (Fig. 2a) is attributable to deformation due to drying. The oxygen content also increased as image brightness increased. However, it was not possible to determine the phase of the mineral. The relatively high C and N content measured in the mineralized areas might reflect the integration of proteins, including collagens. The large Si peak is from the silicon nitride (SiN) film-window substrate (Fig. 1a bottom). The results confirm that CaP mineralization can be imaged in aqueous solution by ASEM.

### CaP crystallization imaged *in vitro* by ASEM

**Figure 3:**
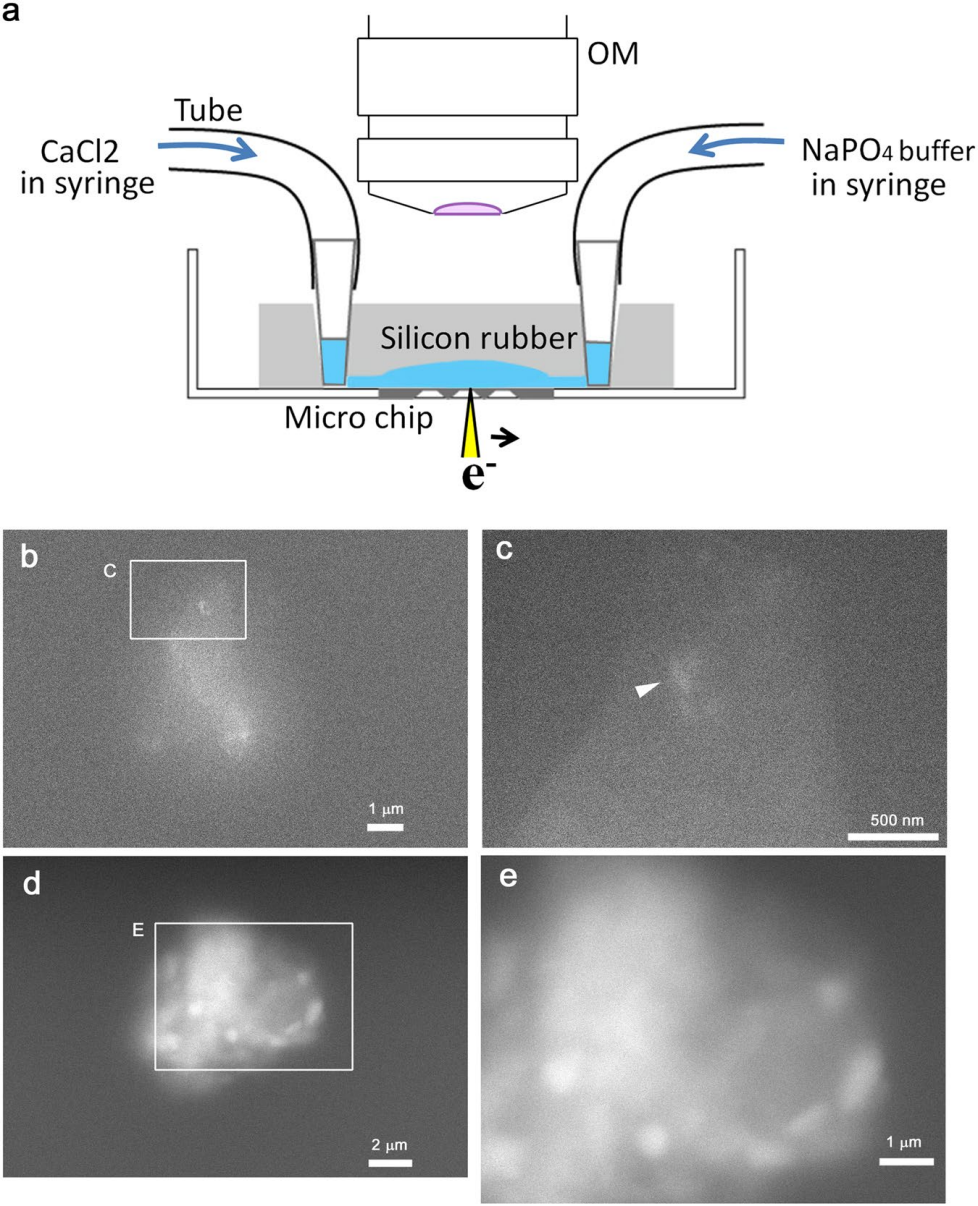
Inorganic crystal formation in crystallization chamber imaged by ASEM. Crystals were inorganically formed by merging 0.2 M CaCl_2_ and 0.2 M PB (pH 7.4) solutions in a microfluidics chamber on the ASEM dish. (**a**) Microfluidics chamber designed to observe CaP crystal formation. (**b**) CaP crystals and ACP formed between CaCl_2_ and PB (pH7.4) solutions in the chamber. (**c**) Higher magnification image of the square in (**b**). (**d**,**e**) Crystals and ACP in a bulk mixture of CaCl_2_ and phosphate buffer. A bulk mixture of 2.5 mM CaCl_2_ and 1 mM PB (pH7.4) was incubated for 5 days, and centrifuged quickly. The precipitate was resuspended in a small aliquot of the supernatant solution, placed on the ASEM dish, and observed by ASEM. (**d**) Low magnification image of a window. (**e**) Higher magnification image of the square in (**d**).

To follow the formation of simple calcium phosphate crystals in aqueous solution by ASEM, we designed and fabricated a small volume-microfluidics chamber with two inlets on the ASEM dish; the chip with SiN windows formed the base of the chamber (Fig. 3a). Approximately 2 μl of CaCl_2_ solution and 2 μl of sodium phosphate buffer (PB, pH 7.4) were added to the inlet lines, and parts of the volumes were simultaneously introduced to the chamber with glow-discharged SiN windows, so that the interface formed above the windows. This was aided by OM observation from the top (Fig. 3a). After 30 seconds, many fine dots were detected in the region where the CaCl_2_ and PB (pH 7.4) solutions started to mix. At higher magnification, the dots were imaged as ambiguous densities and crystals as small as 50 nm in width (Fig. 3b,c). The ambiguous density was interpreted as amorphous calcium precipitates (ACP) that can be transformed to a crystalline phase.

To visualize crystal growth in a bulk mixture, 0.75 ml of 2.5 mM CaCl_2_ solution and 0.75 ml of 1 mM PB (pH 7.4) were mixed and centrifuged at 18000 × g at room temperature (RT). The resulting precipitate was resuspended in a small volume of the supernatant solution, and a small aliquot of the suspension was placed on the SiN windows of the ASEM dish and imaged by ASEM (Fig. 3d,e). Elongated CaP crystals (100 nm–1 μm) were observed amongst ambiguous density that was presumably ACP. Together, the results suggest that the technique can image crystallization in real-time for smaller and larger precipitates.

### NCMIR and PTA staining visualized cell organelle surrounding CaP mineralization in osteoblast cultures

**Figure 4:**
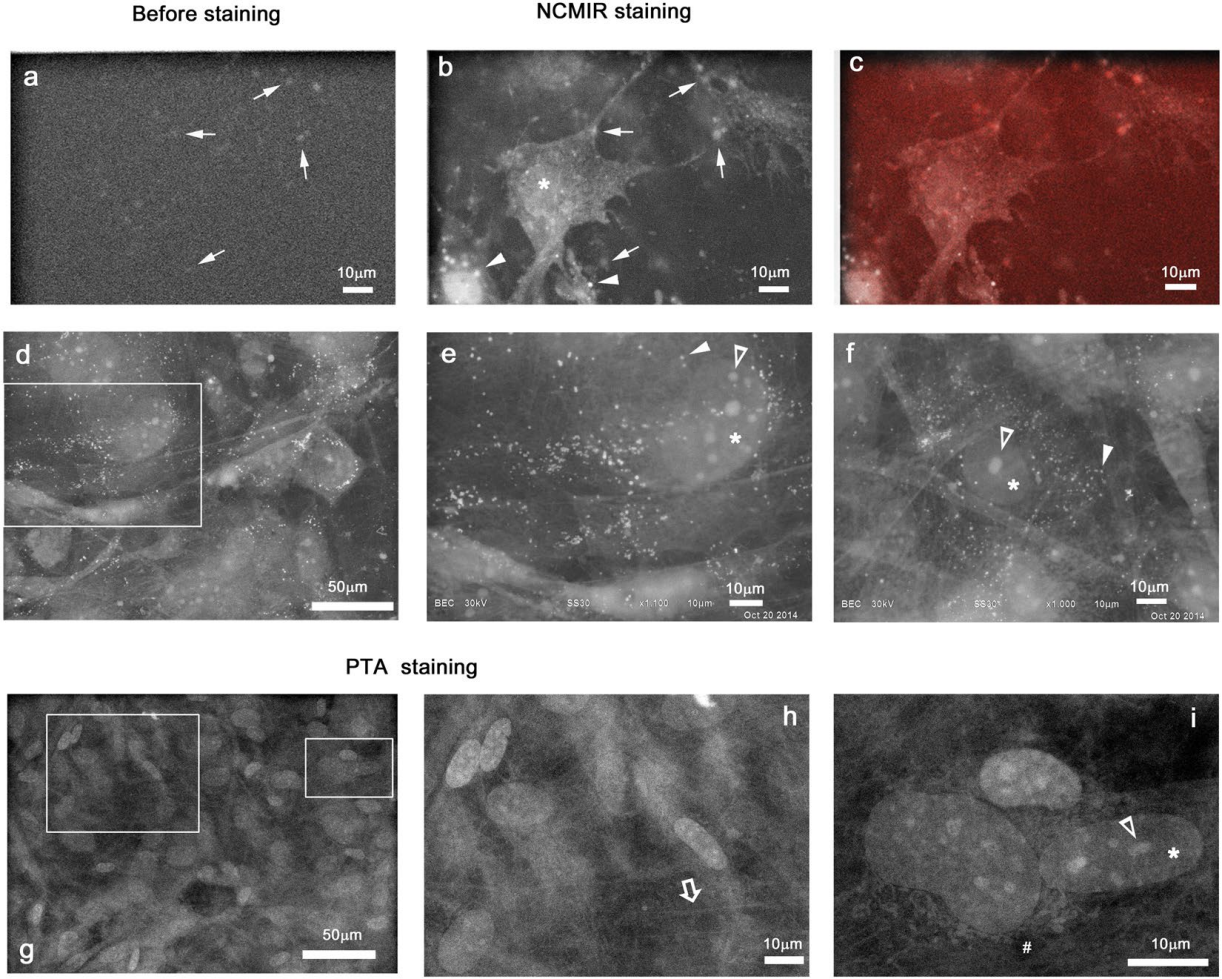
Structures surrounding mineralization in osteoblast primary culture visualized by metal staining. (**a**) Mineralization imaged without staining using ASEM. (**b**) The same region counter stained by the NCMIR method to reveal surrounding structures^29,44^. Osteoblast cells extending filopodia are clearly visible. Their nucleus (*) is imaged in bright shades. The mineralization is also clearly visible as bright spots (arrows). The very bright spots that appeared on counter staining might be oil droplets (arrowheads). (**c**) Overlay image of (**b**) and red-colored (**a**). The mineralized spots are imaged red. (**d**) Cell-dense area stained by the NCMIR method. (**e**) Higher magnification image of the square in d. (**f**) Another area stained by the NCMIR method. (**g**) PTA-stained cell-dense area. (**h**,**i**) Higher magnification images of the squares in g. Filamentous structures (open arrow) were imaged outside the osteoblasts. Their nucleus (*) sometimes included the clear nucleoli (open arrowheads).

On DID10, osteoblast primary cultures were fixed with GA, and examined by ASEM before (Fig. 4a) and after staining (Fig. 4b) by metal solution. Imaging precisely the same region twice, revealed mineralization alone and together with cellular structures in the proximity. The National Center for Microscopy and Imaging Research (NCMIR) staining method moderately stained membraneous structures, nuclei (*) and fine intracellular structures as well as filopodia, and strongly stained round structures (arrowheads) in the cytoplasm (Fig. 4b). In a more cell dense area, the NCMIR method again strongly stained round structures (arrowheads) around the nucleus sometimes including clear nucleoli (open arrowheads) (Fig. 4d–f). From their shape and distribution, these (arrowheads) could be oil droplets, that are reported to be present in COS7 cells^21^ and fat liver tissue^31^.

In contrast, phosphotungstic acid (PTA) staining methods known to stain proteins and nucleic acids, stained nuclei, nucleoli and intracellular structures (Fig. 4g–i). The membranous structures around nucleus (#) might be mitochondria (Fig. 4i), that are reported for cultured cells using ASEM^31^. The brightly imaged extracellular filaments (Fig. 4h, open arrow) might be collagen fibers. These results indicate that PTA or NCMIR staining can be employed to specifically stain subcellular structures surrounding mineralization, but have different staining preferences. For the following tissue observation, we adopted PTA staining which preferably stains proteins.

### ASEM observation of bone tissues before and after staining

Since mouse bones begin to mineralize around embryonic day 15 (E15), mineralization was expected to be observed at later timepoints without staining. After confirming that the mineralization-negative control, mouse liver, exhibited very faint signals under the imaging conditions used, bones were directly imaged in solution by ASEM.

### Spongy bone of Talus

**Figure 5:**
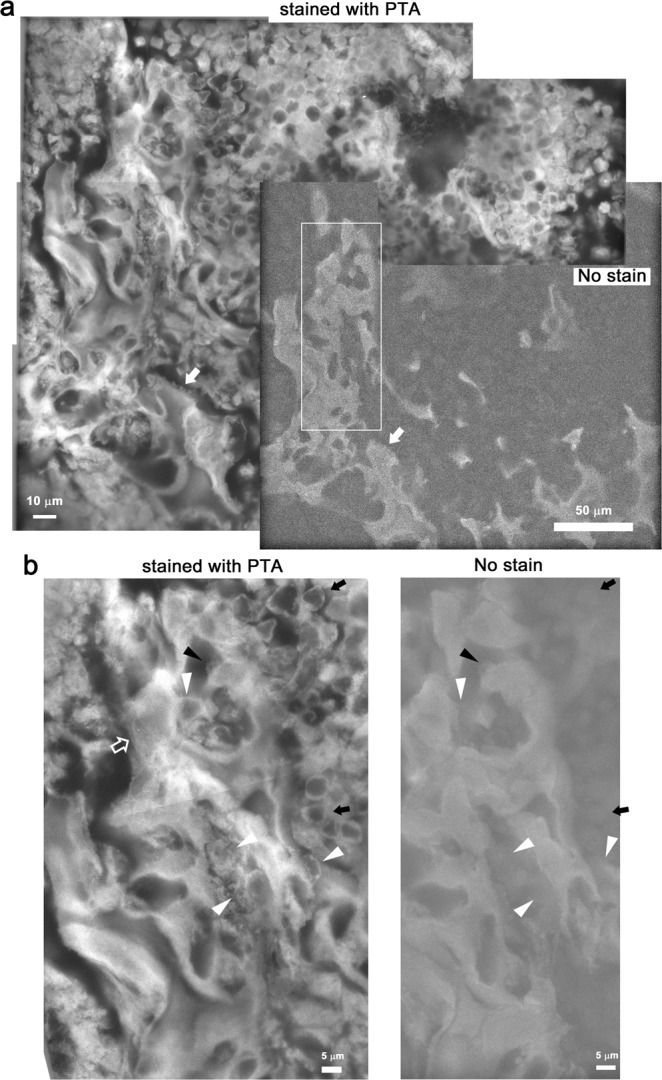
Spongy bone of talus imaged with and without PTA staining by ASEM. Talus was fixed with GA, isolated, sliced into 200 μm thick slabs, and directly imaged in glucose buffer by ASEM. (**a**) Collage of low magnification images of a slab region before (bottom right) and after PTA staining (left and top right). The white rectangle marks a trabecula with high electron density (white arrow). (**b**) Higher magnification images of the white square in (**a**) before (right) and after PTA staining (left). Larger cells attached to the trabecula that were hardly visible before staining, might be osteoclasts (white arrowheads). In contrast, smaller cells that were only faintly visible before staining, might be osteoblasts (black arrowheads). The abundant filaments (open arrow) of trabecula might be collagen 1 fibers. Both panels are a collage of images.

**Figure 6:**
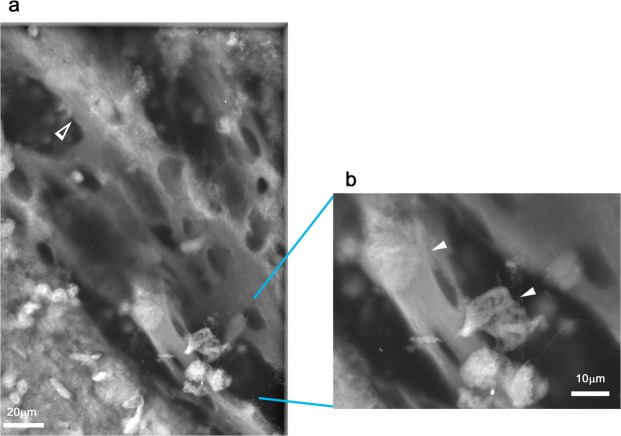
Another spongy bone in talus stained with PTA and imaged by ASEM. Another area of trabecula bone imaged using ASEM. (**a**) Low magnification image collage showing the bone after PTA staining (left). Trabecula is indicated by an open arrowhead. (**b**) Higher magnification image of the indicated area in the left panel. A bright trabecula with many holes has cells that are larger than 10 μm attached to it. These might be osteoclasts (arrowheads).

A new born mouse (P0) talus was fixed using GA in cacodylate buffer, cut along the longitudinal axis using a vibratome, and the section face was placed on the SiN film of an ASEM dish (Fig. 1a, right path). The unstained tissue slab was immersed in glucose buffer and inspected to find a well mineralized area. This was achieved using the inverted SEM, by sliding the tissue on the SiN film window aided by OM observation of the unstained tissue from the top^28^. Well-calcified areas were examined at a range of magnifications. An example is shown in Fig. 5. In the images, dark chambers are separated by bright walls (Fig. 5a bottom right, white arrow) that are interpreted as mineralized trabeculae of spongy bone. Afterwards, the sample was stained with PTA and the same area was observed using ASEM (Fig. 5a top left). Higher magnification images reveal differently sized cells attached to the mineralized trabeculae (Fig. 5b). Round cells that exhibited only a faint signal before staining are prominent around the trabeculae (Fig. 5b black arrows) and might be haematopoietic cells in the bone marrow. Large cells hardly visible before staining observed in some small clefts of the trabeculae, either attached to them or separated from them by a slight gap, might be osteoclasts (Fig. 5b white arrowheads). Smaller cells only faintly visible before staining, are attached to the bright bone and might be osteoblasts (black arrowheads). Fine filaments close to some walls might be collagen fibers (Fig. 5b, open arrow). Another spongy bone region of the same talus slab was similarly observed (Fig. 6). Cells larger than 10 μm were attached to bright trabeculae with many holes, and are considered to be osteoclasts (Fig. 6b, white arrowheads).

### Cortical bone of tibia

**Figure 7:**
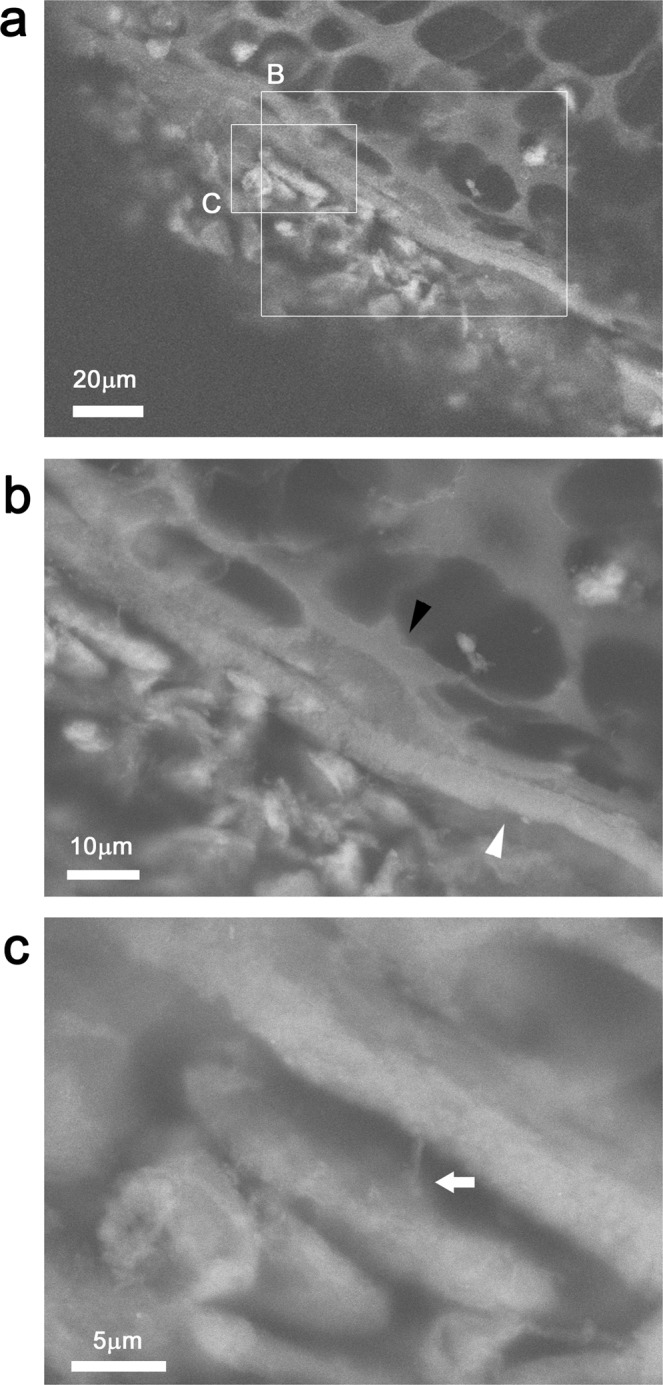
Cortical bone of tibia stained with PTA and imaged with ASEM. Tibia was stained with PTA and the cortical bone near the joint connecting the tibia to the femur was imaged using ASEM. Bone runs diagonally in the micrographs; the region closest to the joint is top left and the region furthest from the joint is bottom right. (**a**) Low magnification. (**b**,**c**) Higher magnification of the annotated squares in panel (a). The images reveal cortical bone layer (white arrowhead) lined on the inner side by many chamber-like structures isolated by homogeneous bright partitions (black arrowhead). (**c**) Some cells close to the outer side of the cortical bone have thin connections to the bone (arrow).

Cortical bone, i.e., compact bone, forms the hard outer shell of most bones in our body and bears much of the hard structure of the skeleton. Mouse tibia was sectioned into 200 μm slabs, stained with PTA, and observed using ASEM. To observe developing cortical bone, we first found spongy bone of the tibia and then shifted the observation area towards the proximal articular joint between the tibia and the femur. At the outer edge of the cartilage tissue, a highly dense continuous thick envelop was observed, i.e., cortical bone (Fig. 7). On the inner side, the bone was lined by many chamber-like structures isolated by homogeneous bright partitions (Fig. 7, black arrowhead). The chambers closest to the cortical bone were more elongated (flatter), suggesting that they are hypertrophic chondrocytes. The outerside of the cortical bone was mainly surrounded by connective tissue. At higher magnification, the layered structure of cortical bone included many fine grains (Fig. 7b,c, white arrowhead). Cortical bone was sometimes attached to cells via thin protrusions (Fig. 7c arrow) extending from the cell body, suggesting an interaction between cells and bone.

### Distribution of cathepsin K in spongy bone using immuno-gold labeling

**Figure 8:**
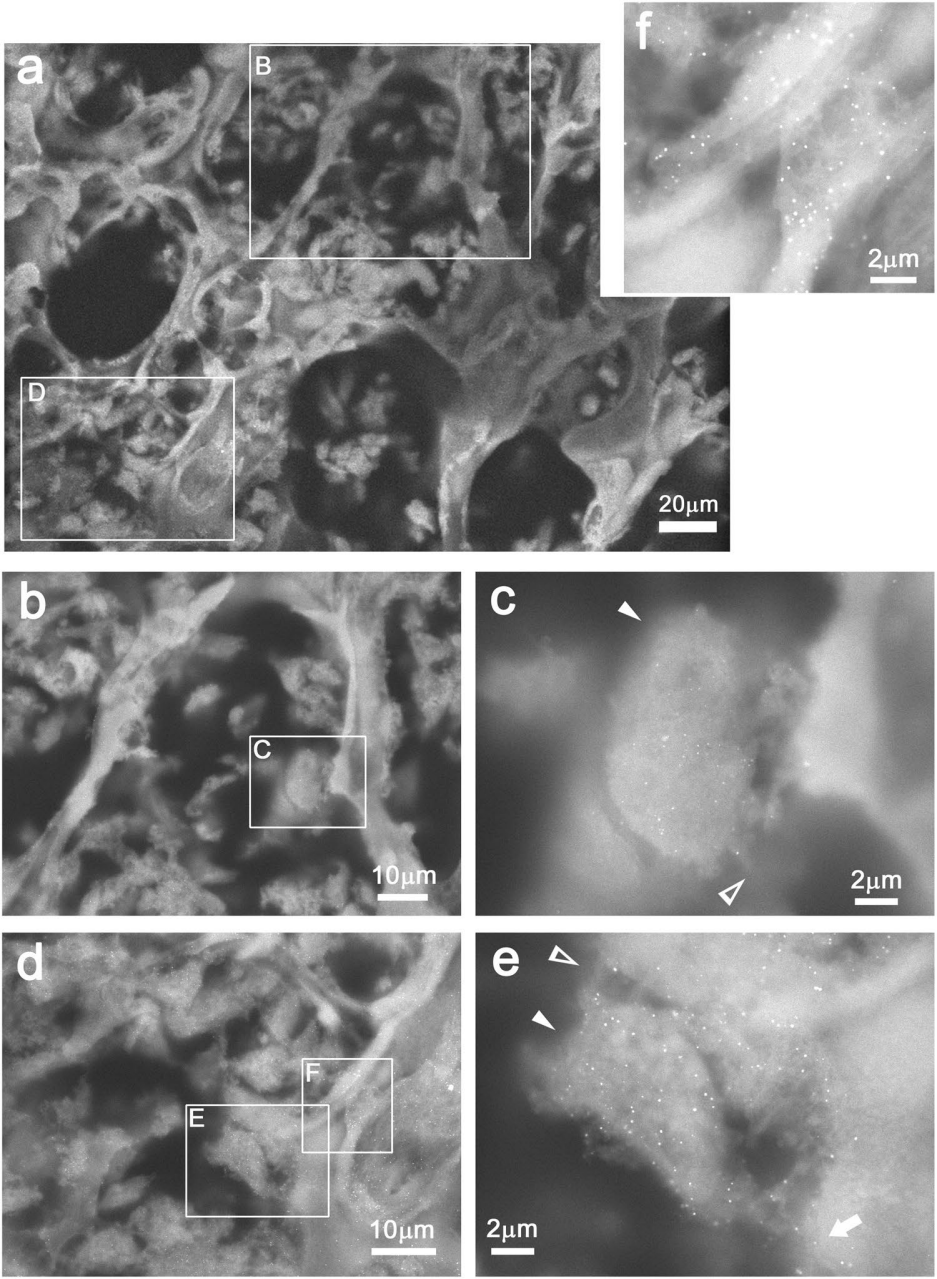
Distribution of cathepsin K in femur spongy bone in solution using immuno-gold labeling and ASEM. Femur thick slabs were labeled with anti-cathepsin K antibody and further with secondary antibody conjugated with FluoroNanogold, gold-enhanced, and then counter-stained with PTA. (**a**) ASEM image of spongy bone. Trabeculae are clearly observed. (**b**–**f**) Higher magnification of the annotated white rectangles indicated in the preceding panels. (**c**) A cathepsin K positive cell attached to a thin trabecula. Gold signals are observed as strong bright dots. (**d**) Another area in (**a**) was observed at higher magnification. Trabeculae are surrounded by many cells of various shape. (**e**,**f**) Higher magnification images of the annotated rectangles in (**d**). (**c**,**e**) A cathepsin K positive cell (arrowheads) that covers a pit of thin trabeculae. In both cases, many substances that were probably digested or secreted by the cell were observed between the cell and the trabecula (**c**,**e**). Gold signals also observed on the trabeculae (arrow) might correspond to cathepsin K secreted from osteoclast cell. (**f**) Cathepsin K positive cells attached to trabeculae.

To identify the large cells attached to bone, spongy bone tissue was immuno-labelled for the osteoclast marker cathepsin K and imaged by ASEM. Cathepsin K protease secreted by osteoclasts degrades bone collagen and thus plays a critical role in bone absorption^32^. Femur slabs, 200 μm thick, were labeled with anti-cathepsin K antibody and further with a secondary antibody conjugated to FluoroNanogold, and gold-enhanced. Afterwards, the slabs were counter-stained with PTA. ASEM images showed that trabeculae in the spongy bone were surrounded by and attached to many cells of various shape (Fig. 8) as in Figs 5 and 6. Higher magnification revealed that large cells attached to the thin trabecula walls had many small bright dots, i.e., gold particles, indicating that they are cathepsin K positive (Fig. 8c,e,f arrowheads); such signals were hardly detected in the corresponding areas of a negative control made without primary antibody (Supplementary Fig. 2). Moreover, because the cathepsin K-positive cells sometimes extended filopodia (open arrowheads) and attached to trabeculae displaying light or heavy mineralization, they are interpreted as osteoclasts. Some of the cathepsin K-positive cells covered a pit of thin mouse trabeculae, with the result that many probably secreted and/or digested substances were observed between the cell and the trabeculae (Fig. 8c,e). The trabeculae themselves were also sometimes partly immuno-labelled (arrow), in good agreement with the observation that cathepsin K secreted from osteoclasts associates with bone.

## Discussion

Here, we demonstrate that CaP mineralization can be observed in aqueous solution at atmospheric pressure by ASEM. Using bone tissue from newborn mice (Fig. 5), we were able to monitor the early stages of mineralization by ASEM. EDS showed that the bright signals in the ASEM images arose from structures containing Ca, P and O (Fig. 2). Since the sample pretreatment required for ASEM is quick, consisting of medium changes of aldehyde solution followed by washing at one stage, fine mineralization is only minimally affected.

We also successfully observed mineralization and the surrounding soft cells and matrices in cortical- and spongy- bone tissues immersed in aqueous solution using ASEM (Figs 5–8), avoiding the time-consuming decalcification-Epon embedding required for TEM. The ASEM method has three main advantages for tissues. First, hard tissue, such as bone, can simply be cut using a sharp scalpel. The two new surfaces resulting from this single cut can then be imaged by ASEM. For hard tissues and some skins, this is much easier than cutting 2–8 μm-thick slices for OM or 70–200 nm-thick Epon slices for TEM. Second, sample pretreatment is short, simple and performed in an aqueous environment. The time required can be limited to the time it takes the reagents, e.g., fixatives, to penetrate the sample to a depth of a few micrometers and interact, because the specimen thickness observable using the inverted SEM is just 2–3 μm at an acceleration voltage of 30 kV^23^. Third, the high-resolution SEM imaging (ca. 8 nm) and single-section preparation, allow bone to be assessed for microcracks with minimum perturbation.

Sudden fracture of bone in spite of sufficient bone mass is suspected to be caused by the accumulation of microcracks^33^, altered collagen crosslinking^34^ and spatial variance of mineral and other components as well as other factors. Microcracks are known to have various causes. One is the physical stress bone is exposed to when osteoporosis patients are treated with the osteoclast inhibitor bisphosphonate (BP)^33^. In this case, microcrack accumulation is attributable to reduced remodeling activity^35^ and/or to the abnormal HA alignment caused by direct BP binding^36^.

qBSE/qBEI techniques using SEM also do not require thin sections and can quantify the mineralization in the surface of an embedded thick tissue. In ASEM, if part of the tissue is not attached to the SiN film window due to surface roughness, the quantification is not guaranteed, because the electron signal from this region is decreased by the buffer layer between the tissue and the SiN film. The advantage of ASEM is the ability to observe fully wet soft tissues without hydrophobic treatment and embedding.

The observations of inorganic CaP crystallization in aqueous solution using the cell-free microfluidic crystallization system were performed without any pretreatment. The result was consistent with previous reports on calcium phosphate crystallization. The mixed 0.2 M CaCl_2_ and sodium phosphate buffer solution (pH 7.4) is supersaturated with respect to amorphous calcium phosphate (ACP), octacalcium phosphate and HA^37^. In supersaturated calcium phosphate solutions, mineralization includes three steps: formation of prenucleation clusters, the aggregation of clusters to form ACP, and transformation from ACP to a crystalline phase^37–39^. The second and third steps are associated with densification previously revealed by dynamic light scattering^38^ and cryo-TEM techniques^39^. The observations shown in Fig. 3b,c can be attributed to the formation of ACP and subsequent densification.

The cell-free microfluidic CaP crystallization system developed here (Fig. 3a) could be also used as a simplified *in vitro* model to investigate abnormal HA alignment, possibly via time-lapse imaging, because live-imaging of CuSO_4_ crystal growth in the crystallization solution has already been demonstrated at 17 frames/sec^40^. For this purpose, additional inlets for possible crystallization modulators, including BP, should be developed to realize real time monitoring of abnormal HA alignments. The new dual inlet chamber developed here would also allow various kinds of inorganic mineralization and crystal formation to be studied.

While mineralization can be observed directly in the ASEM without staining, heavy metal stains or other labeling procedures are required to visualize less electron dense material such as protein (Figs 4–8). PTA generally stains proteins and chromatins, while NCMIR stains lipids, membranes and proteins^21,28,29,31^. Importantly, the hydrophilic environment in the ASEM dish preserves the antigenicity of proteins allowing them to be identified and localized by immuno-gold labeling; the immuno-gold labeling of various proteins has been demonstrated for different cell cultures^22–26,41^. Here, immuno-EM of tissue in solution was realized for the first time (Fig. 8). It determined the localization of cathepsin K and identified osteoclasts in mouse femur. The open configuration of the ASEM dish and the OM above, is advantageous. It facilitates targeting the immuno-labeled area by ASEM and allows operations to be carried out on the tissues during observation, e.g., fine position adjustment by sliding the tissue on SiN film window. Repeated shifting-imaging cycles allow the observation area to be increased as demonstrated^28^. Immuno-EM in solution should now be extended to other molecules important for the bone-metabolism and –remodeling, particularly to investigate protein-mineralization relationships, including the bridging of aged collagen by advanced glycation end products (AGEs) that leads to abnormal HA alignment^34^.

For the elementary analysis of CaP minerals by EDS, we did not coat the sample because a metallic conductive coating (e.g. platinum) would make interpreting the P signal challenging. In the analysis, CaP minerals were observed, but their phase was not determined. To overcome the limitations of the method, the combination of ASEM with a different technique, such as Raman spectroscopy in aqueous solution, should be investigated in future studies.

Many potential applications of ASEM have a high clinical relevance. For example, it could be employed to study the osseointegration of bone implants. Artificial bone, hip-joint implants and dental implants have been intensively developed using metal, surface-roughened metals and various kinds of non-metallic materials. High-throughput ASEM could be used to image the interface between such materials and tissue, allowing the degree of osseointegration to be directly assessed. For this, the osteoblast culture could be combined with the fabrication of micro metal platforms on the SiN film window of an ASEM dish. The latter technique was developed and employed to make electrodes and study electrochemical processes at the molecular level by ASEM^40^.

As already demonstrated, ASEM can be used to precisely image bacterial biofilms^26,41^. This is important, because decalcification is known to be highly related to biofilm formation^42^. Biofilm formation is also an important factor in periodontitis, a major cause of tooth loss^42^. Based on the results presented here, ASEM could be applied to study the formation of such biofilms and their relationship to the osteogenesis and gum inflammation that are symptoms of this widespread dental condition. Biofilm formation by calcite-precipitating bacteria (e.g., *Sporosarcina pasteurii*) and its mineralization^43^ is also a fascinating target for ASEM.

The rapid, high-throughput ASEM method that uses ‘single-cut’ preparations rather than thin sections, is also a potential research tool for mineralization-related diseases including osteoporosis and chondrocalcinosis. Since ASEM successfully visualized haematopoietic cells in spongy bone (Figs 5 and 8), it could be applied to study haematopoiesis and the immune system in bone marrow. In an earlier study, ASEM also quickly identified breast cancer cells metastasized to the lung and spinal cord, using their larger nucleus as a cancer marker^28^. Since definitive diagnosis of bone cancer is carried out using bone biopsy and OM, the single cut method made possible by ASEM in combination with or without immuno-labeling could be applied to investigate osteosarcoma and leukemia, and for the intraoperative diagnosis of primary bone cancers and various cancers metastasized to bone.

## Conclusions

The rapid ASEM method outlined and demonstrated here, allows osteoblasts and bone tissues to be imaged in aqueous solution at atmospheric pressure. The single section protocol is useful to observe hard cortical bone and delicate spongy bone. Additional staining using different metal solutions visualized bone metabolism-related cells and large protein complexes as well as haematopoietic cells immersed in natural aqueous buffer. The new dual inlet chamber developed here allowed formation and visualization of CaP crystals as small as 50 nm using ASEM. It would also allow the effect of additional reagents on CaP mineralization to be investigated, and the study of CaP and other inorganic mineralization and crystal formation processes to reveal the dynamics of crystal growth. Quick immuno-labelling method of tissues should be applied together with in-solution ASEM to study bone-metabolism and -remodeling, to study teeth and related diseases and to other fields, including the diagnosis of various bone-related diseases and other diseases, such as cancers.

## Methods

### Animals

For osteoblast primary culture and tissue preparation, C57BL/6J mice were sacrificed. The methods and the experiments were conducted with the approval of the Animal Research Committee according to the regulations on animal experimentation at National Institute of Advanced Industrial Science and Technology (AIST), Nagasaki University and Kyoto University.

### Primary culture of osteoblasts

Primary cultured osteoblasts were prepared^30^ and analyzed^30,31^ as described previously. In each case, 5 Culture dishes were fixed at 3, 5, 8 and 10 days after the induction of osteoblast differentiation. Detailed information of the culture is given in the Supplementary Methods.

### Tissue sample preparation

Post-natal day 0 C57BL/6 mice (Riken Bioresource Center, Tsukuba, Japan) were euthanized and fixed in 4% PFA in 0.1 M cacodylate buffer (CB, pH7.4) at RT overnight. Tissues were excised from the organ, further fixed with 4% PFA in CB at RT for 15 min and sliced with a PRO7 linear slicer (Dosaka, Kyoto, Japan) to obtain 200 μm thick slabs^27^, or cut manually using a scalpel for immuno-labeling. For PTA staining, the excised tissue was further fixed with 2.5% GA in CB at RT for 2 h^28^ and sliced similarly.

### Staining

Fixed tissues were stained with 2% PTA (TAAB Laboratories Equipment Ltd., Aldermaston, UK) for 3 h at RT for ASEM as described previously^27^. Cells observed by inverted SEM, were counter-stained using PTA or by the NCMIR staining method^29,44^ developed by the Ellisman group^44^. Detailed information of the NCMIR staining is given in the Supplementary Methods.

### Immuno-gold labeling

Immuno-gold labeling of cathepsin K of bone tissue was performed as described^23,45^. Detailed information of the labeling is given in the Supplementary Methods.

### ASEM imaging

ASEM images were recorded using the ClairScope ASEM system (JASM-6200; JEOL Ltd., Tokyo, Japan) as described previously^21^. Briefly, the standard 35 mm bio-ASEM dish with eight 100-nm thick (250 × 250-μm) SiN film windows was employed to observe samples in aqueous liquid^28^. A fixed tissue slab was laid cut face down on the SiN film of the ASEM dish, immersed in a buffer containing 10 mg/ml (w/v) glucose as radical scavenger and 1 mM CB (pH 7.4) and 60 mM KCl, and observed using the inverted SEM as described^31^. The fixed osteoblast cultures were imaged in 10 mg/ml (w/v) glucose solution using the ASEM. The acceleration voltage of the inverted SEM was 20 kV for osteoblast cultures and 30 kV for bone tissues and inorganic mineralizations. Backscattered electrons were recorded by a backscattered electron imaging (BEI) detector (Fig. 1a) for visualization. The electron dose applied for biological samples was less than 0.43 e^−^/Å^2^, which is less than 1% of the dose of 47 e^−^/Å^2^ permitted in low-dose cryo-EM aiming at atomic-resolution single-particle reconstructions. The penetration depth was 2–3 μm at an accelerating voltage of 30 kV, and slightly less (around 2 μm) at an accelerating voltage 20 kV^23^.

### EDS-SEM

EDS of cultured osteoblasts was carried out as described previously^30^. Briefly, after observation in aqueous solution using the inverted SEM, osteoblast cells on the ASEM dish were dehydrated using an ethanol series and dried. The specimen on the chip was mounted on the aluminum holder with adhesive carbon tape, and the uncoated specimen was examined in an FE-SEM (JSM 7400F, JEOL) equipped with an EDS (EDAX Genesis, Ametec) at an acceleration voltage of 20 kV.

## Supplementary information

Dataset 1
