# Supplementary material for: Calcium phosphate mineralization in bone tissues directly observed in aqueous liquid by atmospheric SEM (ASEM) without staining: microfluidics crystallization chamber and immuno-EM
Source: Sci Rep. 2019 May 14;9:7352. doi: 10.1038/s41598-019-43608-6 (PMC6517404; doi:10.1038/s41598-019-43608-6)
Supplement: Supplementary file 1 — Dataset 1 [file 41598_2019_43608_MOESM1_ESM.docx]

Supplementary Information

**Calcium phosphate mineralization in bone tissues directly observed in aqueous liquid by atmospheric SEM (ASEM) without staining: microfluidics crystallization chamber and immuno-EM**

Chikara Sato^1*^, Daiju Yamazaki^2^, Mari Sato^1^, Hiroshi Takeshima^2^ Nassirhadjy Memtily^1, 3^, Yuri Hatano^1^, Takayuki Tsukuba^4^ and Eiko Sakai^4^

^1^ Biomedical Research Institute, National Institute of Advanced Industrial Science and Technology (AIST), Central 6, Higashi 1-1-1, Tsukuba, Ibaraki 305-8568, Japan.

^2^ Graduate School of Pharmaceutical Sciences, and Graduate School of Medicine, Kyoto University, Yoshida Shimo Adachi, 46-29 Sakyo, Kyoto 606-8501, Japan.

^3^ Traditional Uyghur Medicine Institute of Xinjiang Medical University, 393 Xinyi Rd, Urumqi, Xinjiang Uyghur Autonomous Region, 830011 China.

^4^ Division of Dental Pharmacology, Department of Developmental and Reconstructive Medicine, Nagasaki University Graduate School of Biomedical Sciences, 1-7-1 Sakamoto, Nagasaki 852-8588, Japan

* **Correspondence** should be addressed to Chikara Sato, ([ti-sato@aist.go.jp](mailto:ti-sato@aist.go.jp)).

**Supplementary methods**

**Primary culture of osteoblasts**

Primary cultured osteoblasts were prepared as described^1^. Briefly, osteoblast precursors were liberated from neonatal calvaria by treatment with 1 mg/ml collagenase (Wako Pure Chemical) and 2 mg/ml dispase (Godo Shusei, Japan), and tissue debris was removed by filtration through a 160-µm mesh (Tokyo Screen, Japan). The cells were isolated and then grown in a proliferation medium containing 10% fetal calf serum (FCS). After reaching semi-confluency, osteoblasts were re-plated to ASEM dishes in a medium containing 100 ng/ml BMP-2 (R&D Systems) and 10% FCS, to stimulate cellular differentiation. The osteoblasts were cultured for 10 days (DID10), unless indicated otherwise (Fig.1b-c, Supplemental Fig. 1a-b). The cells were fixed with 4% paraformaldehyde (PFA; Wako Pure Chemicals, Osaka, Japan) in phosphate-buffered saline (PBS; 136 mM NaCl, 1.4 mM KCl, 10 mM Na_2_HPO_4_, and 1.7 mM KH_2_PO_4_, pH 7.4) at RT for 15 min, and further fixed with 2.5% GA in PB (pH7.4) at RT for 15 min for ASEM.

**NCMIR Staining method**

NCMIR staining was conducted as described^2,3^. Briefly, osteoblasts were washed with 0.15M cacodylate buffer (CB, pH7.4) containing 2 mM CaCl_2_, and fixed and stained with the same buffer supplemented with 1.5 % potassium ferricyanide (Sigma) and 2% aqueous osmium tetroxide (OsO_4_) (Nisshin EM) at 25 °C for 10 min. After washing with double distilled water (DDW), cells were then incubated with filtered 1 % thiocarbohydrazide (Tokyo Chemical Industry) at 25 °C for 20 min. After rinsing with DDW, they were then further fixed and stained with 2 % aqueous OsO_4_ at 25 °C for 5 min, rinsed with DDW, stained with 2 % uranyl acetate in DDW, and incubated at 4 °C overnight. Finally, after washing with DDW, cells were stained with 0.4 % lead citrate (TAAB Laboratories Equipment) at 25 °C for 2 min.

**Immuno-gold labeling**

Immunolabeling of cathepsin K^4^ was basically performed as described^5,6^. For the fixed tissues, non-specific binding was blocked using 5% bovine serum albumin, 10% normal goat serum, 1% fetal bovine serum in PBS. For primary labeling, tissues were incubated with rabbit anti-cathepsin K antibodies^5^ (1:2000 dilution) added to the blocking solution. For secondary labeling, tissues were incubated with goat antibodies: Fab’ against rabbit IgG, doubly conjugated with 1.4 nm Nanogold® and fluorescent Alexa Fluor® 488 dye (FluoroNanogold^TM^, Nanoprobes) (1:250 dilution in the blocking solution). The bound antibodies were fixed with 1% GA in PBS for 15 min at room temperature. After washing with double distilled water (DDW), Nanogold particles were enhanced by gold sedimentation using GoldEnhance EM (Nanoprobes) at room temperature for 5 min.

**
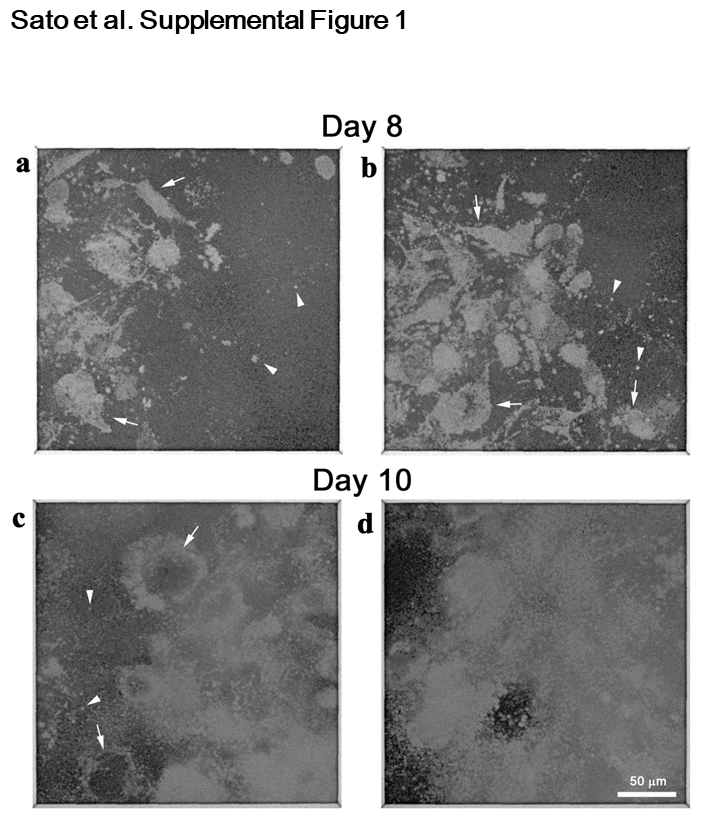
**

**Supplemental Figure 1. Electron dense sedimentations in osteoblast primary culture imaged by ASEM.**

Osteoblasts cultured on the ASEM dishes were GA-fixed at DID8 or DID10, and observed by ASEM as in Fig.1b-d. (a-b) On DID8, small bright signals appeared, forming cell shapes (arrows) surrounded by fine grains (arrowheads). (c-d) Later time points; the number of bright signals increased as the culture proceeded.


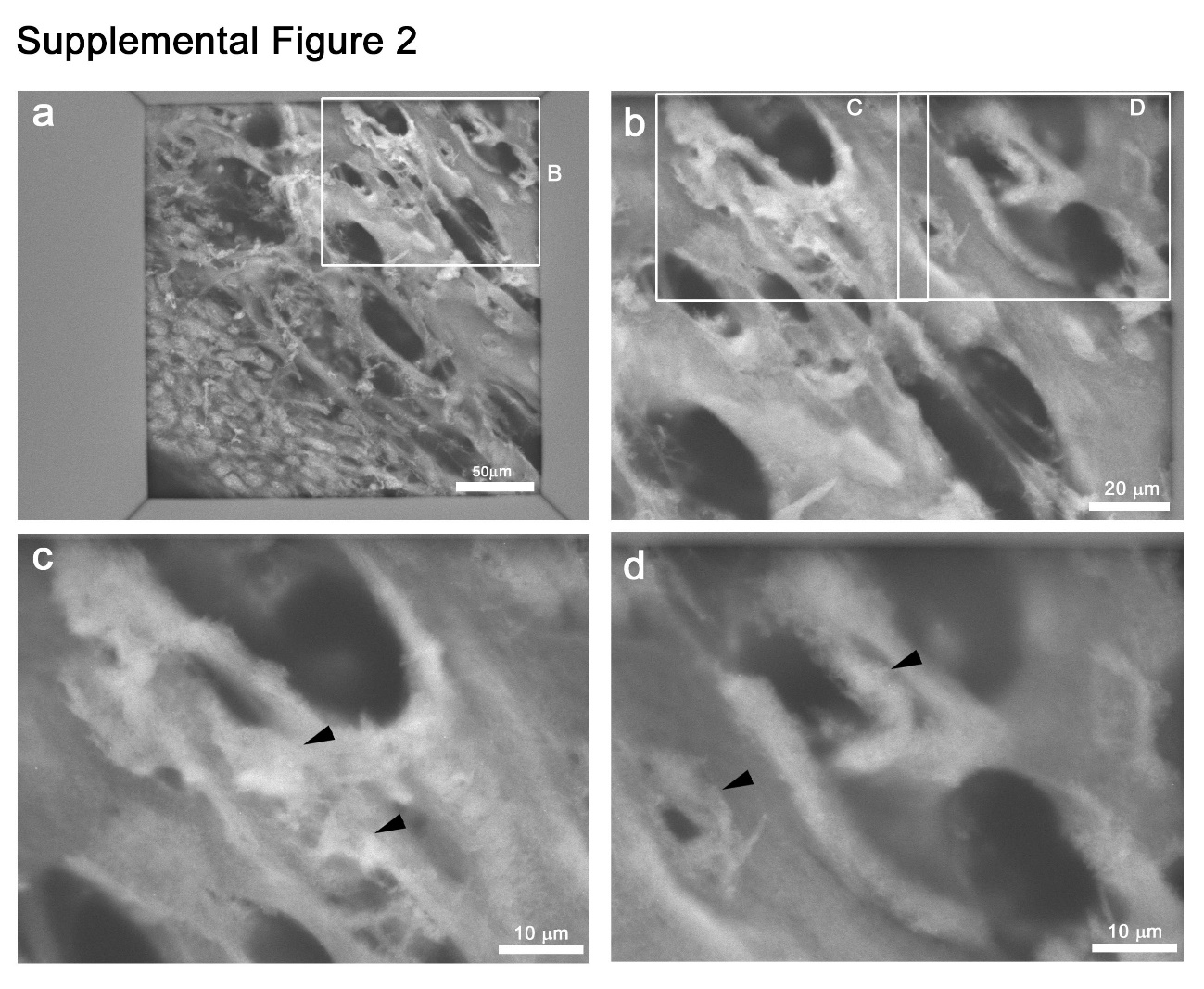


**Supplementary Figure 2. Negative control of immuno-gold labelling of femur spongy bone without primary antibody using ASEM.**

In these control experiments, femur thick slabs were labeled with FluoroNanogold-conjugated anti-rabbit Fab’ secondary antibody alone, gold enhanced, and stained with PTA. (a) ASEM images of spongy bone in femur. (b) Higher magnification image of the white rectangle B in (a). (c, d) Higher magnification images of the white rectangles C and D in (b), respectively. Hardly any signal was detected when only the FluoroNanogold-secondary antibody was used, i.e., without the primary antibody.

**References**

1 Zhao, C. *et al.* Mice lacking the intracellular cation channel TRIC-B have compromised collagen production and impaired bone mineralization. *Sci Signal* **9**, ra49 (2016).

2 Deerinck, T. J., Bushong, E. A., Thor, A. & Ellisman, M. H. NCMIR methods for 3D EM: a new protocol for preparation of biological specimens for serial blockage scanning electron microscopy. *Microscopy*, 6-8 (2010).

3 Itoh, K. *et al.* Mucin-type core 1 glycans regulate the localization of neuromuscular junctions and establishment of muscle cell architecture in Drosophila. *Developmental Biology* **412**, 114–127 (2016).

4 Bromme, D., Okamoto, K., Wang, B. B. & Biroc, S. Human cathepsin O2, a matrix protein-degrading cysteine protease expressed in osteoclasts. Functional expression of human cathepsin O2 in Spodoptera frugiperda and characterization of the enzyme. *The Journal of biological chemistry* **271**, 2126-2132 (1996).

5 Yamaza, T. *et al.* Study of immunoelectron microscopic localization of cathepsin K in osteoclasts and other bone cells in the mouse femur. *Bone* **23**, 499-509 (1998).

6 Maruyama, Y., Ebihara, T., Nishiyama, H., Suga, M. & Sato, C. Immuno EM-OM correlative microscopy in solution by atmospheric scanning electron microscopy (ASEM). *J Struct Biol* **180**, 259-270 (2012).
